# Structural equation modelling of work related musculoskeletal disorders among dumper operators

**DOI:** 10.1038/s41598-023-40507-9

**Published:** 2023-08-28

**Authors:** Mohith Bekal Kar, Mangalpady Aruna, Bijay Mihir Kunar

**Affiliations:** Department of Mining Engineering, NITK Surathkal, Mangaluru, India

**Keywords:** Risk factors, Mechanical engineering

## Abstract

The aim of this study is to investigate the impact of personal factors, habitual factors, and work-related factors on work-related musculoskeletal disorders (WRMSDs) among dumper operators. In total, 248 dumper operators working in an iron ore mine were considered for this study. A questionnaire was developed and administered to collect dumper operators' personal, habitual, and work-related data. The reliability of the questionnaire was cross-checked by Cronbach alpha and the test–retest method. The values of Cronbach alpha for all latent variables were above 0.7, and the correlation coefficient of the questionnaire items at Time 1 and Time 2 was above 0.82. After verifying the validity (i.e., convergent and divergent) of the questionnaire data, the relationship between the factors under consideration was examined by structural equation modeling (SEM). The SEM demonstrated a moderate fit, with $\frac{{\upchi }^{2}}{df}$ value of 1.386, comparative fit index (CFI) of 0.86, goodness-of-fit index (GFI) of 0.72, adjusted goodness-of-fit index (AGFI) of 0.69, Tucker-Lewis Index (TLI) of 0.83, normed fit index (NFI) of 0.71 and root mean square error of approximation (RMSEA) of 0.051. The SEM analysis revealed a positive relationship between WRMSDs and personal factors (with path coefficient = 0.313 and p < 0.05) as well as work-related factors (with path coefficient = 0.296 and p < 0.05). However, the relationship between WRMSDs and habitual factors was not statistically significant (with path coefficient = 0.142 and p > 0.05). Overall, this study provides valuable insights into the factors that influence the prevalence of WRMSDs among dumper operators. The findings highlight the significance of personal and work-related factors by which one can make a positive decision to prevent and reduce the incidence of WRMSDs among dumper operators.

## Introduction

The demand for the raw resources, such as minerals, has risen globally due to urbanization, increasing demand for finished goods and technological innovations^1^. To meet this demand, the mining industry relies heavily on Heavy Earth Moving Machinery (HEMM), such as drills, excavators, transport equipments, and auxiliary equipments. These machines are essential for the efficient extraction and transportation of minerals.

In surface mines, dumpers are commonly used to transport waste and minerals. Dumper operators face several challenges in their daily work, such as—need to sit for extended period^2^, exposure to whole-body vibration^3,4^, and adopting poor posture while driving^5^. These factors contribute to the development of Work-Related Musculoskeletal Disorders (WRMSDs) among dumper operators^6^.

WRMSDs is a serious problem for both the operators and the mining industry. The study conducted by the Indian Council of Medical Research (ICMR) showed that 65% of the workers in the mining industry suffers from WRMSDs^7^. The economic burden of WRMSDs is significant, with direct and indirect costs estimated to be billions of dollars annually^8^. In addition, WRMSDs constitute a significant cause of disability among workers and loss in productivity in many industries worldwide^9^.

Dumper operators, in particular, are at risk of developing WRMSDs due to the physically demanding nature of their work^10^. They drive and maneuver heavy vehicles on rough terrain for loading and unloading material repetitively throughout the day^11^. The prevalence of WRMSDs among dumper operators in Indian surface metal mines is poorly understood, and further research is needed to identify the risk factors associated with WRMSDs.

Several risk factors may contribute to the development of WRMSDs among dumper operators, such as individual characteristics—age and body mass index^12,13^, work-related factors—job demand and job control^14^, the physical environment of the mine, the ergonomics design of the dumper truck, and management practices^13^. Recent studies have identified the risk factors associated with WRMSDs among mine workers^15–18^. These studies have adopted various study designs, were multiple dependent variables (such as lower back pain, neck pain, etc.) are combined to form a new and single dependent variable for the analysis^19,20^. However, the complex relationships between multiple risk factors and multiple outcomes cannot be studied using this aproach. Advance statistical techniques, such as structural equation modeling (SEM) is useful in understanding complex relationships between exposures and health outcomes^21^. In addition, SEM can model the latent constructs that may not be directly measurable^22^. Therfore, in this study, SEM technique was used to analyse the relationship between personal, habitual, and work-related factors with respect to WRMSDs among dumper operators working in Indian surface iron ore mines.

## Results

### Reliability of the questionnaire

The reliability of the custom questionnaire was determined using the Cronbach alpha and test–retest methods. The Cronbach alpha test determines the internal consistency of the latent variable, whereas the test–retest method determines whether the data collected is independent of time.

#### Cronbach alpha test

The internal consistency of the latent variables, including personal, habitual, and work-related factors, was assessed using Cronbach's alpha test. For each latent variable, the variance of the scores for each item was calculated to determine the variability in responses. Additionally, the variance of the total scores across all items for each latent variable was also computed. These variances were used in calculation of Cronbach's alpha coefficient using the standard formula. The result indicated alpha coefficients of 0.82, 0.76, and 0.77 for personal, habitual, and work-related factors. The high coefficient of 0.82 for personal factors shows that the responses to the items related to personal factors are highly correlated and it measures the same underlying construct. Though the coefficient of habitual factors is less than that of personal factors, it is considered acceptable^23^. Similarly, the coefficient of 0.77 for work-related factors is also considered acceptable. Therefore, the questionnaire developed in this study has adequate reliability and can be considered for the study.

#### Test–retest method

The stability and consistency of the custom questionnaire over time were assessed by re-administering it to a subset of the sample after a 9-month interval. A total of 20% of the participants were selected to participate in the retest. The questionnaire response collected at Time 1 was compared with the response collected at Time 2. The result indicated a strong (ranging from 0.82 to 0.91) and statistically significant correlation between the responses to the items of the custom questionnaire at Time 1 and Time 2.

### Validity of the questionnaire

#### Convergent validity

Table 1 shows the correlation matrix that demonstrates the relationship between the observed variables. The results indicated in Table 1 reveals a moderate but significant correlation among the variables belonging to personal factors (i.e., age, experience in mines, number of childrens, eduction and marriage status). Similar trend was also observed among the variables belonging to the habitual (i.e., tobacco chewing, medicine intake, smoking cigarettes, alcohol consumption) and work-related factors (i.e., awkward posture, repetitive work, job demand, work design).

**Table 1:**
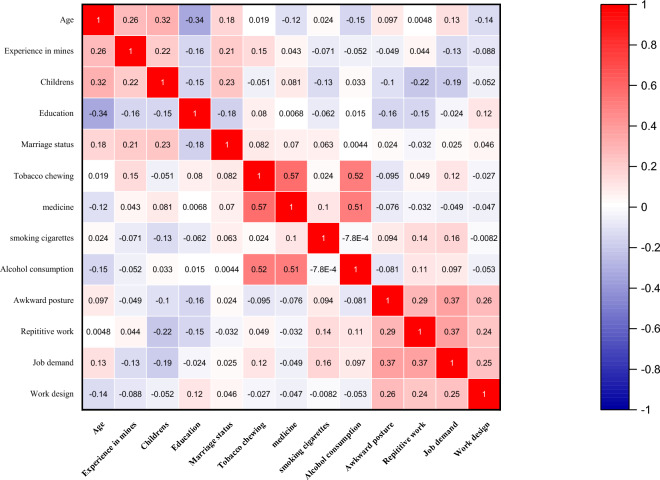
Correlation matrix representing the inter-variable relationships.

#### Discriminant validity

The discriminant validity of the latent variables such as personal, habitual, and work-related factors, was found using correlation coefficient. The results revealed that all independent latent variables had correlation coefficients less than 0.2 and were statistically insignificant (*p* > 0.05). This suggested that there is minimal or no relationship between the latent variables.

### Factor analysis

The exploratory factor analysis (EFA) was done to explore the underlying factor structure of the custom questionnaire. The factor structure obtained from the EFA was in line with the proposed factor structure developed using the theoretical background. The confirmatory factor analysis (CFA) was then performed to confirm the fit of the observed data with the hypothesized model.

**Figure 1:**
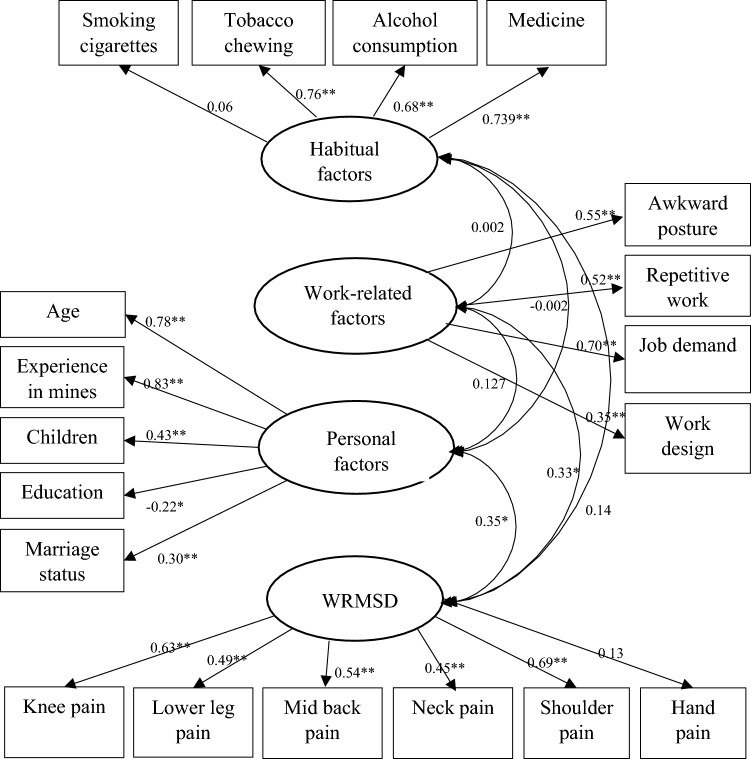
Pictorial representation of CFA model. *p < 0.05 and **p < 0.001.

The CFA showed that the fit of the model is moderate^23^ with $\frac{{\upchi }^{2}}{df}$ value of 1.386, comparative fit index (CFI) of 0.86, goodness-of-fit index (GFI) of 0.72, adjusted goodness-of-fit index (AGFI) of 0.69, Tucker-Lewis Index (TLI) of 0.83, normed fit index (NFI) of 0.72 and root mean square error of approximation (RMSEA) of 0.056. It is worth to mention here that there was a significant increase in the fit of the model when the indicator variables, such as “smoking cigarettes” and “hand pain” were removed from the model. However, the evaluation of the SEM is more objective while retaining these two indicator variables. Hence, these indicator variables were retained and the final model comprises of four latent variables and ninteen indicator variables as shown in Fig. 1.

### Structural equation modelling

**Figure 2:**
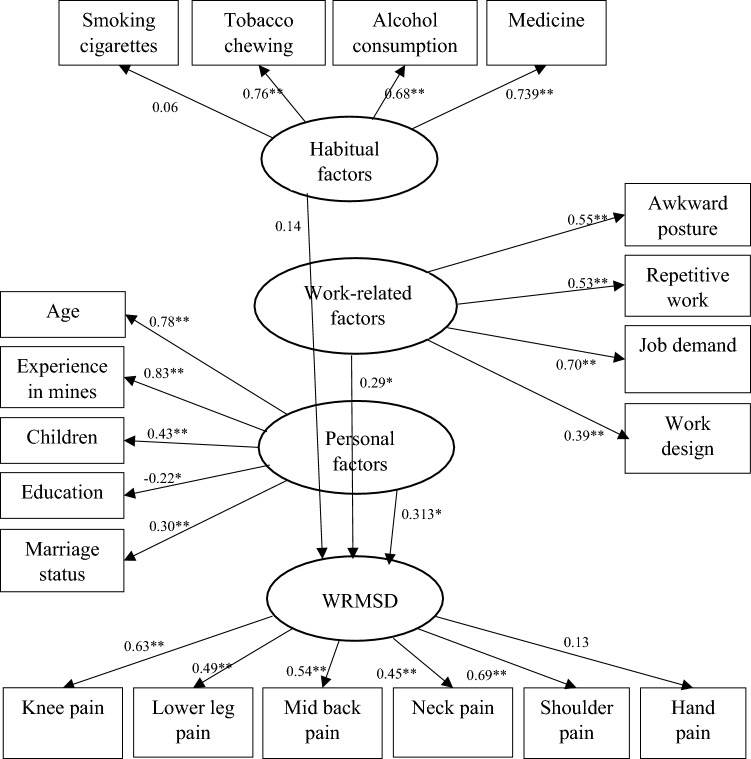
Pictorial representation of SEM. *p < 0.05 and **p < 0.001.

The relationship between the variables were examined by structural equation modelling (SEM) using the generalized least squares method for parameter estimation. The final SEM (as shown in Fig. 2), demonstrated a moderate fit, with $\frac{{\upchi }^{2}}{df}$ value of 1.386, CFI of 0.86, GFI of 0.72, AGFI of 0.69, TLI of 0.83, NFI of 0.71 and RMSEA of 0.051. The SEM analysis revealed the relationship between the latent independent variables (i.e., personal factors, habitual factors, and work-related factors) and dependent variables (i.e., WRMSDs). The path coefficient between the “personal factors” and ‘age’ was 0.78 (*p* < 0.001), indicating a strong and positive correlation. A strong and positive relationship was also found between “personal factors” and “experience in driving” (path coefficient = 0.83 with *p* < 0.001). A similar pattern was noticed for the indicator variables such as "number of children" (path coefficient = 0.43, p < 0.001) and "marriage status" (path coefficient = 0.30, p < 0.001). However, the path coeffcient between “personal factors” and ‘education’ was weak and negative (path coefficient = − 0.22 with *p* < 0.05).

Similarly, “habitual factors” had a strong and positive relation with indicator variables such as, “alcohol consumption” (path coefficient = 0.687 with *p* < 0.001), ‘medicine’ (path coefficient = 0.739 with *p* < 0.001), and “tobacco chewing” (path coefficient = 0.767 with *p* < 0.001). In addition, “work-related factors” had a moderate and positive relation with the “awkward posture” (path coefficient = 0.551 with *p* < 0.001), “job demand” (path coefficient = 0.703 with *p* < 0.001), and “repetitive work” (path coefficient = 0.528 with *p* < 0.001). However, present study showed a positive and moderate relation between the “work-related factors” and “work design” (path coefficient = 0.393 with *p* < 0.001).

The path coefficients between ‘WRMSDs’ and five indicator variables such as, “knee pain”, “lower leg pain”, “mid-back pain”, “neck pain”, and “shoulder pain” were found significant (with *p* < 0.001) with the path coefficients of 0.632, 0.496, 0.541, 0.452, and 0.695, respectively. Further, when the path coefficient between the latent variables were compared, it was found that ‘WRMSDs’ and “personal factors” had a moderate and positive correlation (path coefficient = 0.313 with *p* < 0.05). In contrast, a weak correlation was found between ‘WRMSDs’ and “work-related factors” (path coefficient = 0.296 with *p* < 0.05). However, the relationship between ‘WRMSDs’ and “habitual factors” was not found to be statistically significant (path coefficient = 0.142 with *p* > 0.05).

The findings of this study partially supported the hypothesized model. The influence of personal and work-related factors on WRMSDs were significant, with *p* < 0.05. This indicates that both these latent factors do affect the prevalence of WRMSDs in dumper operator’s population. On the other hand, present study showed that the WRMSDs was not influenced by habitual factors of the dumper operators, as *p* > 0.05.

## Discussion

This study aims to evaluate the association between personal, habitual, and work-related factors with the WRMSDs among dumper operators. The authors used a custom-made self-reported questionnaire to collect the data on dumper operators. The reliability of the questionnaire was cross-checked by Cronbach alpha and the test–retest method. The values of Cronbach alpha coefficent for all latent variables were above 0.7, and the correlation coefficient of questionnaire items at Time 1 and Time 2 was above 0.82. The CFA was then conducted to assess the fit of the model. Initially, the model had a moderate fit. There was significant increase in the fit of the model after removing the indicator variables, such as “smoking cigarettes” and “hand pain”. However, these indicator variables were retained in the final model because they made the evaluation of the SEM model more objective. The final model comprised four latent and ninteen indicator variables that derived an moderate fit. This rigorous assessment of the questionnaire ensured that the conclusions drawn from the analysis were reliable and valid. The SEM was then used to examine the relationship between the latent variables and the WRMSDs. The path coefficients were computed for all the latent variables. The coefficient values showed that the personal and work-related factors significantly and positively influence WRMSDs, while habitual factors have poor influence.

This study's findings partially align with the previous studies performed on various occupational groups. For example, previous study^24^ showed that personal factors, such as age, gender, and BMI, were significantly associated with the prevalence of WRMSDs. Similarly, one more study^25^ found that work-related factors, such as job demand, job control, and work-related stress, were significantly related to the prevalence of WRMSDs among dumper operators. However, the findings of this study revealed that the habitual factors will not significantly influence the prevalence of WRMSDs among dumper operators, which is not corroborated with the results of previous studies. For example, previous study^26^ found that unhealthy habits, such as smoking, alcohol consumption, and sedentary behavior significantly influence the risk of injuries among mine workers.

Overall, this study provides valuable insights into the factors that influence the prevalence of WRMSDs among dumper operators. The findings highlight the significance of personal and work-related factors, by which one can take a positive decision to prevent and reduce the incidence of WRMSDs among dumper operators. In the future, the findings of this research can be further strengthened by considering a more diverse sample from different mine sites and incorporating additional measures of reliability and validity to evaluate the results.

## Methods

### Background of the mine

The iron ore mine under study covers an area of 62 Ha and had a production capacity of 6 Mt per year. The mine was working in two shifts of 8 h each. The ore was transported from the pit to the dumping point using dumpers of 30 tons capacity. The distance between the loading and unloading point was about 1 km. The dumper operators were performing an average of 11 to 12 cycles per shift (one cycle comprises loaded truck traveling from the loading point to the unloading point and empty truck back to the loading point).

### Data collection

The case study mine employed 262 dumper operators. The inclusion criteria for selecting the study sample were that the operators must have a minimum age of 18 and a maximum age of 56. In addition, operators must have a minimum of 6 months of professional driving experience. Similarly, operators with a history of injuries were excluded. In total, 248 dumper operators were selected, and the information about their characteristics (i.e., personal, habitual, and work-related) was collected using developed questionnaire. Similarly, the WRMSDs data of the study sample were collected using a standardized Nordic questionnaire^27^. Table 2 gives the consolidated values of the collected data.

**Table 2:** Characteristics of dumper operators.

	Variable	Response	Mean ± SD or n (%)
Personal factors	Age		38.47 ± 7.65
	Height		1.71 ± 0.066
	Weight		76.5 ± 10.13
	BMI		25.9 ± 3.06
	Experience in mines		21.62 ± 6.32
	Number of childrens		1.06 ± 0.3
	Education	No formal education	10 (4)
		Primary education	101 (40.3)
		Secondary education	101 (40.3)
		Tertiary education	36 (14.5)
	Marriage status	Single	48 (19.5)
		Married	192 (78)
		Divorced	6 (2.4)
Habitual factors	Medicine	Yes	72 (29.3)
		No	194 (70.7)
	Smoking cigarette	No	206 (83.7)
		Yes	40 (16.3)
	Alcohol consumption	No	172 (69.9)
		Yes	74 (30.1)
Work-related factors	Job demand	No	176 (71.5)
		Yes	70 (28.5)
	Work design	No	220 (89.4)
		Yes	26 (10.6)
	Repetitive work	No	172 (69.4)
		Yes	74 (29.8)
	Awkward posture	No	224 (90.3)
		Yes	22 (8.9)

### Data analysis

The data collected from the self-reported questionnaire was analyzed by SEM, which was coded using semopy python library^28^. The parameter estimate of the SEM model was determined by the least square estimation method. The goodness of fit of the SEM model was assessed by seven types of fit indices: chi-square statistic, GFI, AGFI, NFI, TLI, CFI, and RMSEA. The SEM is considered to have an acceptable fit if the chi-square statistic is not significant (i.e., *p* > 0.05), the RMSEA is less than 0.08, and othet fit indices such as GFI, AGFI, NFI, TLI, and CFI are greater than 0.90^23^.

### Ethical considerations

Ethical approval for this study was obtained from the institutional review board (committee set by National Institute of Technology Karnataka). All methods were performed in accordance with the relevant guidelines and regulations set by the institutional review board. The participants were informed about this study, and consent was obtained from them. Confidentiality of the participant’s personal and medical information was ensured.

## Data Availability

The data sets analysed during this study are not publicly available due to specific terms and conditions set by the mine under study. However, upon reasonable request, the data can be made available from the corresponding author in normalized/standardized form.
